# MicroRNA dysregulation in spinal cord injury: causes, consequences and therapeutics

**DOI:** 10.3389/fncel.2014.00053

**Published:** 2014-02-25

**Authors:** Manuel Nieto-Diaz, Francisco J. Esteban, David Reigada, Teresa Muñoz-Galdeano, Mónica Yunta, Marcos Caballero-López, Rosa Navarro-Ruiz, Ángela del Águila, Rodrigo M. Maza

**Affiliations:** ^1^Molecular Neuroprotection Group, Experimental Neurology Unit, Hospital Nacional de Parapléjicos (Servicio de Salud de Castilla-La Mancha)Toledo, Spain; ^2^Departamento de Biología Experimental, Facultad de Ciencias Experimentales y de la Salud, Universidad de JaénJaén, Spain; ^3^Unidad de Patología Mitocondrial, Unidad Funcional de Investigación en Enfermedades Crónicas, Instituto de Salud Carlos IIIMadrid, Spain

**Keywords:** spinal cord injury, microRNA, nervous system, cell death, inflammation, astrogliosis, therapeutics

## Abstract

Trauma to the spinal cord causes permanent disability to more than 180,000 people every year worldwide. The initial mechanical damage triggers a complex set of secondary events involving the neural, vascular, and immune systems that largely determine the functional outcome of the spinal cord injury (SCI). Cellular and biochemical mechanisms responsible for this secondary injury largely depend on activation and inactivation of specific gene programs. Recent studies indicate that microRNAs function as gene expression switches in key processes of the SCI. Microarray data from rodent contusion models reveal that SCI induces changes in the global microRNA expression patterns. Variations in microRNA abundance largely result from alterations in the expression of the cells at the damaged spinal cord. However, microRNA expression levels after SCI are also influenced by the infiltration of immune cells to the injury site and the death and migration of specific neural cells after injury. Evidences on the role of microRNAs in the SCI pathophysiology have come from different sources. Bioinformatic analysis of microarray data has been used to identify specific variations in microRNA expression underlying transcriptional changes in target genes, which are involved in key processes in the SCI. Direct evidences on the role of microRNAs in SCI are scarcer, although recent studies have identified several microRNAs (miR-21, miR-486, miR-20) involved in key mechanisms of the SCI such as cell death or astrogliosis, among others. From a clinical perspective, different evidences make clear that microRNAs can be potent therapeutic tools to manipulate cell state and molecular processes in order to enhance functional recovery. The present article reviews the actual knowledge on how injury affects microRNA expression and the meaning of these changes in the SCI pathophysiology, to finally explore the clinical potential of microRNAs in the SCI.

## INTRODUCTION

Spinal cord injury (SCI) is a dyscapacitating pathology with a global-incident rate estimated at 23 new traumatic SCI cases per million in 2007 (180,000 new cases/year; Lee et al., 2013). Recovery, even partial, from SCI was considered unachievable until the early 1980s when David and Aguayo (1981) demonstrated that spinal axons could regenerate under appropriate conditions. This seminal work initiated a burst of research aimed to understand the pathophysiology of the SCI and the development of therapeutic tools. The capacity of microRNAs to regulate cell state and function through post-transcriptionally silencing hundreds of genes are being acknowledged as an important actor in the pathophysiology of SCI. In this article, we review the known and potential roles of microRNAs in SCI and their possible therapeutic application. The article begins with an overview of the pathophysiology of SCI and the gene programs involved, followed by a general picture of microRNA biogenesis, function, and regulation, particularly in the central nervous system (CNS). Afterward, we discuss the observed global changes in microRNA expression following SCI and the role of mechanisms regulating microRNA biogenesis on SCI. We next explore the contribution of microRNA in the regulation of key processes of SCI such as inflammation, cell death, regeneration, or gliosis. The review ends with a brief perspective on the feasibility of microRNA-based therapeutics in the treatment of SCI.

## AN OVERVIEW OF THE PATHOPHYSIOLOGY OF THE SPINAL CORD INJURY

The term spinal cord injury refers to the damage to the spinal cord caused by trauma or disease but also to the physiological response that develops after injury and that largely determines the functional deficits that will face the injured person. Although most SCI are traumatic, inflicted by mechanical causes due to accidents or violence, there are also a number of non-traumatic SCIs, mainly due to discopathies and tumors (Biering-Sorensen et al., 1990). The pathophysiology of the SCI is a complex intermingled set of events, responses, mechanisms, and processes, affecting the nervous, vascular, and immune systems, that develop during the months following the initial damage. Most participating cells reside in the spinal cord, but others are summoned to the injury site from the circulatory system. A brief description of the pathophysiology is provided in the following paragraphs. Detailed descriptions can be obtained in different reviews (Mautes et al., 2000; Dumont et al., 2001; Profyris et al., 2004; Liverman et al., 2005; Rowland et al., 2008; Oyinbo, 2011).

In the traumatic SCI, the mechanical trauma causes the compression, stretching, laceration, or transection of the spinal cord. The most common form of SCI is a compressive–contusive-type injury in which displaced components of the vertebral column, exert force on the cord causing both immediate traumatic injury and often sustained compression (Rowland et al., 2008). Once compression and contusion surpass structural thresholds, physical and biochemical alterations of the cells induce a cascade of systemic and local events that constitute the primary damage (Oyinbo, 2011). Local events include axon severing, membrane rupture and death of neurons, glia and endothelial cells. Surviving neurons at the injury site respond firing action potentials that shift the local levels of ions together with the ions released due to membrane shear. The resulting ion concentration reaches toxic levels that kill the nearby neurons. The barrage of action potentials also causes the release and accumulation of neurotransmitters that will cause further neuron and glial cell death by excitotoxicity. Mechanical trauma causes intraparenchymal hemorrhage (mainly in the small vessels of the gray substance) and, consequently, the disruption of the blood–spinal cord barrier together with edema and swelling at the spinal cord (Mautes et al., 2000). Vasospasm and thrombosis in the superficial vessels accompany hemorrhage causing hypoxia, ischemia, and increasing neural cell death. At a systemic level, primary damage causes a transient increase in systemic blood pressure that is followed by a prolonged hypotension (either hemorrhagic or neurogenic) causing further oxygen deprival to the spinal cord. Hypoxia – together with ion shifts inside and outside the neuron – seems to cause a temporal switch off of the spinal cord function at and below the injury site known as spinal shock.

In the minutes to months that follow the initial damage, the secondary phase of the SCI takes place. This secondary phase comprises several interrelated damage processes including vascular alterations, biochemical disturbances and cellular responses that lead to an inflammatory response and cell death that significantly expand the area of damage. Vascular alterations resulting from hemorrhage and ischemia are central constituents of the secondary injury cascade. Reduced perfusion of the spinal cord due to vasospasm and hypotension is followed by a period of reperfusion, which increases the production of oxygen- and nitrogen-derived free radicals [superoxide, hydroxyl radicals, nitric oxide (NO), peroxynitrite] already being produced during the period of ischemia (Dumont et al., 2001). All these species contribute to oxidative stress and exacerbates damage and cell death. Alterations in the vascular system also include the disruption of the blood–spinal cord barrier that extends far beyond the injury site for days and even weeks after injury. Release of cytokines [interleukin (IL)-1β, tumor necrosis factor-α (TNF-α)], matrix metalloproteinases, reactive oxygen species (ROS), etc., contribute to enhance vascular permeability (Mautes et al., 2000; Donnelly and Popovich, 2008) and – together with upregulation of cell adhesion molecules (CAMs and selectins) by endothelial cells (Mautes et al., 2000; Profyris et al., 2004; Donnelly and Popovich, 2008) – participate in the recruitment and infiltration of immune cells to the injured spinal cord.

Immune cells develop a key role in the pathophysiology of SCI. Neutrophils arrive to the injury site in the first hours after injury [peaking at 1 day post-injury (dpi) in rats and 1–3 dpi in humans, see Fleming et al., 2006] and disappear during the first week, although some evidences indicate a continued presence for long times (180 dpi in rats, according to Beck et al., 2010). Neutrophils remove debris, but mainly release assortments of proteins, including proteolytic and oxidative enzymes that “sterilize” the area but also contribute to extend tissue damage (Taoka et al., 1997). Neutrophils also release signaling proteins that attract macrophages. Macrophages resulting from the activation of spinal cord microglia or from blood monocytes infiltrate the injury in the first days after the injury, presenting a peak during the first week and persisting for months (Fleming et al., 2006). Microglial activation is triggered early after injury and induces a morphological and functional change in the phenotype of this cell, from a resting, ramified phenotype to a phagocytosis-capable, “macrophage-like” phenotype (Byrnes et al., 2006). Macrophages remove debris and dead cells, present antigens, and release pro-inflammatory and protective cytokines, ROS, NO, and proteases (Fleming et al., 2006). T lymphocytes enter the injured spinal cord mainly 1 week after injury. T cells are responsible for cell-mediated adaptive immunity, although their role in SCI remains controversial (Fleming et al., 2006). In rat models, it seems that immune cells tend to maintain or reduce their presence after this first burst of immune response following SCI. However, a recent study in rats demonstrates that immune cells present a time-dependent multiphasic response, with a late phase that mainly involves a peak of macrophages at 60 dpi (Beck et al., 2010). Contrary to the mixed beneficial and detrimental effects on the immune response in the initial phase, this late phase seems to be mainly beneficial and its blocking causes further functional deficits (Beck et al., 2010).

All previous events have strong effects on neural cells. Necrotic cell death initiated by the mechanical trauma spreads during the secondary phase due to excitotoxicity and the accumulation of free radicals (ROS and RNS) released by immune cells or during reperfusion. Free radicals cause lipid peroxidation as well as oxidative and nitrative damage to lipids, proteins, and nucleic acids, inducing the lysis of the cell membrane, altering the cytoskeleton and the organelles, and ultimately causing the death of neural cells (Oyinbo, 2011). Apoptosis and other forms of programed cell death are also important actors in secondary damage after SCI. Programed cell death seems to occur in at least two phases: an initial phase, in which apoptosis accompanies necrosis and a later phase, which is predominantly confined to white matter and that affects oligodendrocytes and microglia (Profyris et al., 2004). Calcium influx and possibly signaling through Fas/CD95 pathway are among the triggers proposed for programed cell death although other mechanisms may be also acting, including lost of trophic support (Liverman et al., 2005; Rowland et al., 2008).

Apoptosis of oligodendrocytes results in extended demyelination, the loss of the oligodendrocyte myelin sheath that insulate nerve axons and permit effective nervous signal conduction. As a consequence, axons crossing the injured segment/s but deprived from myelin sheath and experiencing alterations in the ion channels become unable to transmit signals to the brain and the body, even though they remain intact. Axotomy (axon sectioning) is also a major factor in SCI. Depending on aspects such as distance of axotomy to cell body, trophic support or neuronal type, the fate of axotomized neurons will differ strongly. A significant proportion of neurons, being severed close to the cell body or lacking enough tropic support, die after axotomy. Others retract the severed axon and develop a terminal retraction bulb. Conversely, some surviving neurons develop a regenerating response. These neurons experience hypertrophy of the cell body due to the increased synthesis of proteins that will be used in an attempt to regenerate the severed axon. Within this pro-regenerative response, intact neurons can also develop sprouts (collateral axonal branches) that may rewire unconnected targets (sometimes with undesired side effects). However, axon growth is strongly if not completely inhibited due to several inhibitors present at the injured spinal cord. Much of this inhibition is due to molecules associated to the oligodendrocyte myelin, such as NOGO, and other are consequence of the changes experienced by the astrocytes following injury. Astrocyte reactivity after SCI involves cellular changes characterized by an initial hypertrophic and a late hyperplasic response (Fawcett and Asher, 1999; Sahni et al., 2010). During the hypertrophic response, reactive astrocytes present a characteristic enlarged somata with thickened processes and upregulation of intermediate filament proteins, such as glial fibrillary acidic protein (GFAP) and vimentin (Pekny and Pekna, 2004). This initial hypertrophic response protects neural cells by up-taking the potentially excitotoxic glutamate, producing oxidative stress scavengers and repairing the blood–spinal cord barrier (Faulkner et al., 2004; Sofroniew, 2005). In the following hyperplastic phase during the subacute and chronic phases of the SCI, newly proliferated astrocytes with thinner processes forms a dense scar in the damaged area that inhibits of axon regeneration (Fawcett and Asher, 1999; Silver and Miller, 2004).

In the months and years that follow the damage, the SCI becomes chronic. Cell death, scarring, gliosis, and other local alterations of the tissue lead to the formation of cavities filled of fluid and surrounded by glial scar that can extend for several segments above and below the injury site (Liverman et al., 2005). Myelin loss and alterations in the functioning of the ion channels can lead to changes in the surviving neurons and dependent networks leading to chronic neuropathic pain and/or spasticity.

## GENE EXPRESSION CHANGES AFTER SPINAL CORD INJURY

Most cell functions, responses, and phenotypic changes depend on the activation and/or suppression of a large number of transcriptional pathways (Di Giovanni et al., 2003). Microarray analyses in SCI models have identified gene expression changes that, to some extent, can be observed across different studies and even across different strains or species (Di Giovanni et al., 2003; Velardo et al., 2004). Prominent expression changes in the spinal cord following injury comprise clustered expression changes in genes associated to:

### TRANSCRIPTION AND STRESS RESPONSE

Clusters including factors such as *NF-κB* (nuclear factor kappa-light-chain-enhancer of activated B cells), *c-fos*, *HSP-70* (70 kD heat shock protein) become upregulated early after injury and continue for at least 24 h (Song et al., 2001). Activation of transcription factors such as NF-κB or interferon regulatory factor (IRF)-1 mediates the increased expression of pro-inflammatory genes and modulates pivotal processes such as apoptosis or regeneration (Song et al., 2001). On the other hand, there is an early increase in chaperone expression (*HSP-70*) together with metallothioneins 1 and 2 (Aimone et al., 2004) that may protect cells from protein damage or oxidative stress. Further anti-oxidative stress genes become upregulated at much later times, including superoxide dismutases (SODs) 1 and 2, *catalase*, and *glutathione peroxidase* (GPX; Aimone et al., 2004).

### INFLAMMATORY RESPONSE

Upregulation of pro-inflammatory genes, including cyclooxygenase (COX)-2, interleukins *IL-1β* and *IL-6*, TNF-α, is observed early after injury and persists during the first weeks to go back to normal levels at 14 days (Carmel et al., 2001; Song et al., 2001; Di Giovanni et al., 2003; Zhang et al., 2004). Chemokines involved in immune cell attraction [monocyte chemotactic protein (MCP)-1, macrophage inflammatory protein (MIP)-1β] and adhesion molecules participating in cell infiltration (integrins, intercellular and vascular CAMs, cadherins, and selectins) are also upregulated during the first hours after injury (Aimone et al., 2004). Changes in the expression of inflammatory genes take place in different spinal cord cells but has been particularly studied in microglia. According to a profiling study developed by Byrnes et al. (2006), activated microglia first express (peak at 4–24 h) pro-inflammatory molecules, including IL-1β, IL-6, CCL2 [chemokine (C–C motif) ligand 2]/MCP-1, CXCL2 [chemokine (C–X–C motif) ligand 2]/M1P2α, involved in the recruitment of immune cells to the damaged area. A second pulse of change in microglial gene expression occurs later (3–7 dpi in rats) and involves genes coding for cytochrome b-245 light chain protein (*CYBa*), cathepsin Y, Galectin-3, microglial response factor (MRF)-1, P38, cyclin D1, caspase 1, and leukocyte surface antigen CD53/OX44, that participate in the regulation of the immune response, phagocytosis, production of ROS, proliferation, and cell death. Other immune response genes related to phagocytosis including the classical complement pathway and the FC receptors show a persistent upregulation after injury (Aimone et al., 2004).

### NEURON-ASSOCIATED GENES

A large cluster of genes coding for proteins involved in the potassium, calcium, and sodium pumps and channels as well as in synapsis, cell excitability, and neurotransmission show a significant decrease during the first week (Carmel et al., 2001; Velardo et al., 2004; De Biase et al., 2005; Wu et al., 2005). This decrease can reflect changes in the gene profile of the neurons but it may also reflect the advance of neuronal cell death that takes place after injury (Carmel et al., 2001; De Biase et al., 2005). Attempts of axonal regeneration in the weeks following injury are also accompanied by expression changes in a large group of genes that includes the overexpression of several plasticity and regeneration-associated proteins (*Ninjurin*, *Coronin 1b*, *Rab13*, *Growth Associated Protein-43*, *Neuritin*, *Ankyrin*, *Myelin oligodendrocyte glycoprotein*, and *cAMP*-related genes; Carmel et al., 2001; Song et al., 2001; Di Giovanni et al., 2003, 2005b).

### CELL CYCLE AND CELL DEATH

Changes in the expression of cell cycle genes has been detected 24 h after injury, including upregulation of *c-myc*, *pcdna*, *gadd45a*, and cyclins (Di Giovanni et al., 2003). Activation of cell cycle genes may induce apoptosis in post-mitotic cells and, thus underlie post-SCI apoptosis of neurons. They may also be involved in astrocytic proliferation during the glial scar formation (Di Giovanni et al., 2003). Pro-apoptotic and anti-apoptotic genes also show significant expression changes, including upregulation of caspase-3, *Bax*, *Bak-1* in the first week after SCI and the later upregulation of protective *PI3K* and *Stat3* and downregulation of pro-apoptotic *GSK-3* (Carmel et al., 2001; Aimone et al., 2004). In addition, it has been observed the upregulation at 24 h of genes coding for different growth factors [transforming growth factor-β (TGF-β), platelet-derived growth factor, vascular endothelial growth factor] and anti-apoptotic proteins (survival of motor neurons proteins), which may contribute to prevent neural cell death (Song et al., 2001).

### VASCULAR SYSTEM REGULATION AND ANGIOGENESIS

Hemorrhage and other early vascular events are reflected in the expression profile. Blood coagulation genes – platelet factor 4 (CXCL4), coagulation factors VIII, protein C, etc., – appear overexpressed in the first 24 h after injury and some remain for several weeks (Chamankhah et al., 2013). Genes such as angiopoietin are increased in the injury area in the week that follows the damage (Aimone et al., 2004). Other dysregulated genes related to vascular events include HIG (hypoxia-induced gene), which is downregulated late after injury (Aimone et al., 2004).

### CHANGES IN GLIAL CELLS

Although less explored in global profile studies, there are important changes in gene expression in the glial cells related to astrocyte reactivity and the formation of the glial scar, as well as to the proliferation and remyelination attempts of oligodendrocytes. Among the best-characterized genes related to astrocyte reactivity and glial scar formation are those coding for GFAP, vimentin, and nestin, intermediate filaments highly overexpressed in reactive astrocytes. These genes are markedly upregulated during the first week after injury (Carmel et al., 2001; Wu et al., 2005). On the other hand, oligodendrocyte profiles are characterized by a decrease in cell specific genes due to the advance of oligodendrocyte death during the first weeks followed by an increase in proliferation and myelination genes (Wu et al., 2005).

## MicroRNA BIOGENESIS, FUNCTION, AND REGULATION

As we have shown in the previous sections, SCI causes profound cellular changes that result from dysregulation of signaling pathways and structural proteins. Alteration of gene expression following SCI is likely accompanied by the post-transcriptional regulation of these modified gene networks. Among the known post-transcriptional regulators, microRNAs have recently attracted much attention due to their ability to inhibit mRNA translation.

MicroRNAs were first identified in *C. elegans* in 1993 (Lee et al., 1993). A small non-coding RNA (lin-4) was shown to regulate translation of lin-14 through RNA–RNA interaction. MicroRNAs constitute an abundant class of highly conserved small non-coding RNA molecules composed of 20–24 nucleotides in length, that post-transcriptional regulate gene expression. More than 2500 mature forms of microRNA sequences have been identified in humans (miRBase; Kozomara and Griffiths-Jones, 2011). MicroRNAs are transcribed from genomic DNA by RNA polymerase II or III in the form of large primary transcripts (pri-miRNAs) with functional secondary structures of stem-loop hairpins. The stem-loops structures are recognized and cleaved by the complex formed by the RNase III Drosha and the RNA-binding protein DGCR8, which leads to liberation of a precursor microRNA (pre-miRNA). Once processed, exportin 5 transports the pre-miRNAs from the nucleus to the cytosol, where an enzymatic complex containing Dicer process them to yield the 20- to 25-nucleotide duplex mature miRNAs. One strand (passenger) of the duplex is degraded and the other strand (guide) is integrated into a RNA-induced silencing complex (RISC), which facilitates the binding of the microRNAs to their target mRNA.

The predominant role of microRNAs in RISCs is to regulate post-transcriptional the expression of their target genes by translational repression, mRNA cleavage, or mRNA decay. The employed mechanism depends on the degree of complementarity between the microRNAs and their target mRNAs. When microRNAs perfectly or near-perfectly pairs the targeted mRNAs, as occurs in plants, cleavage of the mRNA takes place. On the contrary, when microRNAs imperfectly pair to their target mRNAs, as usually occurs in animals, translational repression or decay (due to microRNA-driven deadenylation) become the mechanisms mediating gene regulation. In animals, microRNAs have been proposed to downregulate gene expression mostly through the translational repression (Krichevsky, 2007), although a recent study showed that destabilization (and degradation) of target mRNAs is the major mechanism of miRNA gene repression in mammals (Guo et al., 2010).

Since microRNAs do not require perfect complementarity for target recognition in animals, a single microRNA can regulate multiple, even hundreds mRNAs (Lim et al., 2005; Baek et al., 2008; Selbach et al., 2008). At the same time, each mRNA can be regulated by many microRNAs (Krek et al., 2005; Krichevsky, 2007) and a given microRNA may have multiple binding sites in the same mRNA, thereby enhancing its overall effect (Bhalala et al., 2013). Computational predictions suggest that the more than 2000 known human microRNAs are able to repress the expression of at least a 20–30% of all protein-coding genes (Krichevsky, 2007) and up to the 60% according to Friedman et al. (2009) and Sayed and Abdellatif (2011). Besides a single miRNA can tune protein synthesis from thousands of genes, quantitative proteomics studies have found that the magnitude of the change is small under physiological conditions (less than fourfold; Guo et al., 2010). In fact, blocking microRNA biogenesis through DICER inhibition has modest effects on cell differentiation and organism patterning, although opposite results have also been reported (Kosik, 2010).

MicroRNAs are common components of regulatory pathways, and in many cases constitute molecular on–off switches in establishing cell fate and identity both under physiological and pathological conditions. The power of this regulatory mechanism lies in the unique ability of microRNAs to guide processes and cellular functions through precise titration of gene dosage, and the ability of a single microRNA to control the levels of a large cohort of gene products. Because microRNAs target multiple mRNAs, they can exert distributed control over broad target fields of functionally related mRNAs as opposed to focusing their control on a small number of genes in a “final common pathway.” The action of an individual microRNA can lead to a cumulative reduction in expression of multiple components of one specific functional network, and several microRNAs may cooperatively target various mRNAs whose protein products are part of the same molecular pathway (Krek et al., 2005; Krol et al., 2010; Sluijter, 2013). MicroRNAs are often physically clustered in the genome, and these sets of microRNAs may target mRNAs with related biological functions at short distances in their protein–protein interaction map (Kim et al., 2009). Coordinated microRNA targeting of closely connected genes seems to be prevalent across pathways (Tsang et al., 2010). Thus, microRNAs provide broad and robust transcriptional regulation that can be governed either by individual microRNAs or by the combined action of multiple microRNAs.

MicroRNA networks are often specialized for specific cell types and there is a strong correlation between cell identity and patterns of microRNA expression (Kosik, 2010). The anticorrelated expression of microRNAs and their target mRNAs in developmental transitions and the mutually exclusive expression of target genes and microRNAs in neighboring tissues argues that microRNAs confer accuracy to developmental gene expression programs, thus ensuring tissue identity and supporting cell-lineage decisions, and reflect the basic role of miRNAs in establishing cell identity during development (Ebert and Sharp, 2012). MicroRNAs also serve as a buffer to assist cells in coping with environmental contingencies (Kosik, 2010). As markers of cell identity, miRNAs encode a representation of multiple cell states that all correspond to a single identity. That is, many different states comprise a single identity because cells must retain their identities in the face of both environmental changes and internal noise that can result in large variations in molecular composition. MicroRNAs are good candidates for setting boundary conditions upon coding transcripts to restrict protein levels within a range of values that maintain cell identity in the face of homeostatic compensatory changes. The RISC allows both the constitutive maintenance of cell identity by silencing mRNAs that are not part of the specialized cell’s repertoire as well as the holding of mRNAs of an alternative identity in reserve (Lim et al., 2005). The environment that cells face is many times more complex than the biological adaptations available within the genome. Among the adaptive responses of cells to an environmental contingency is the up- or downregulation of proteins. The properties of miRNAs to adjust protein levels, their dispensability under basal conditions, their conservation, as well as the ease with which new miRNAs appear over evolutionary time all suggest that they are suited for environmental contingencies (Kosik, 2010). Wu et al. (2009) have proposed that miRNAs keep the system close to the mean and set expression boundaries of transcription factors, which are otherwise noisy.

## MicroRNA IN THE NORMAL AND PATHOLOGICAL CENTRAL NERVOUS SYSTEM

MicroRNAs are highly expressed in the mammalian CNS, including the spinal cord (Miska et al., 2004; Kosik, 2006; Krichevsky, 2007; Bak et al., 2008). Their expression in the spinal cord seems to be specific and preserved through vertebrate evolution (Yunta et al., 2012). Moreover, experimental data reveal that some miRNAs are cell-type specific, such as miR-124 and miR-128, which are preferentially expressed in neurons, or miR-23 or miR-219, which are restricted to astrocytes and oligodendrocytes, respectively (Sempere et al., 2004; Smirnova et al., 2005; Lau et al., 2008). MicroRNAs serve essential roles in virtually every aspect of CNS function, including neurogenesis, neural development, and cellular responses leading to changes in synaptic plasticity (Krichevsky et al., 2003; Miska et al., 2004; Sempere et al., 2004; Stefani and Slack, 2008; Gangaraju and Lin, 2009; Li and Jin, 2010; Smith et al., 2010; Cochella and Hobert, 2012; Goldie and Cairns, 2012). For example, experimental overexpression or inhibition of miR-124 have demonstrated its key role in neuronal differentiation (Krichevsky et al., 2006; Makeyev et al., 2007; Visvanathan et al., 2007), whereas let-7b regulates neural stem cell proliferation and differentiation by targeting the stem cell regulator TLX and the cell cycle regulator cyclin D1 (Zhao et al., 2010a). MicroRNAs are also involved in the specification of glia. They have been shown to be critical regulators of oligodendrocyte differentiation and myelination in the vertebrate CNS, in particular miR-219 and miR-338 (Zhao et al., 2010b).

In addition to their role in the development and the functioning in the normal CNS, numerous evidences indicate that microRNA dysregulation is implicated in a wide range of neurological diseases. Several studies indicate that microRNA dysregulation can be associated to neurodegeneration, as in Alzheimer’s disease where the downregulation of the miR-29 cluster has been proposed to contribute to increase BACE1 and amyloid-beta levels and thus to the development of the disease (see Saugstad, 2010). The role of microRNAs in neurodegeneration extends to other pathologies, such as Huntington and Parkinson diseases, or even to prion diseases (Saugstad, 2010). The evidences also suggest that microRNAs participate in psychiatric disorders, such as Schizophrenia, Tourette’s syndrome, and bipolar disorder, as well as in developmental disorders, such as the Rett and fragile X syndromes (Kosik, 2006; Meza-Sosa et al., 2012). MicroRNAs also participate in the profound cellular changes that occur in the damaged CNS, where they play an active role in the regulation of typical features of CNS injuries, such as inflammation, apoptosis, cell proliferation, and differentiation (see this review for SCI or Bhalala et al., 2013 for a more general approach).

## GLOBAL CHANGES IN microRNA EXPRESSION AND REGULATION OF microRNA BIOGENESIS IN SPINAL CORD INJURY

Several studies have analyzed microRNA expression and function in SCI (34 published paper in August, 2013 according to PubMed). Seven of these studies include global analyses of microRNA expression based on microarrays. As shown in **Table 1**, the results from these studies strongly vary in the resulting overall patterns, both in the number (from as few as 10 significant expression changes to more than 250) and profile of changing microRNAs. Much of this variability can be ascribed to differences in SCI model, from ischemia-reperfusion to transection, compression, or contusion, to differences in the sampling time after injury, from 4 h to 14 days after injury, or to differences in the animal model (rats and mice of different strains). Besides, some results from comparable injury models and times still show strong differences. Technical differences among platforms, laboratories, sampling and analytical procedures, etc., may account for variability in microarray data (Git et al., 2010; Callari et al., 2012; Saugstad, 2013). In agreement, we have observed that the number of significant changes identified at 7 dpi vary from 257 to 2 depending on whether parametric (Student’s *t*-test) or non-parametric (rank product test) tests are used (Yunta et al., 2012). However, discrepancies may also reveal variability in microRNA expression associated to differences in injury features. Injury severity is one of such factors. It is well known that severity determines several aspects of the SCI pathophysiology such as the inflammatory response or the nerve fiber preservation/destruction (Fehlings and Tator, 1995; Yang et al., 2005). In agreement, the expression of specific microRNAs (miR-129-2 and miR-146a) has been observed to correlate with functional score after injury, an accepted proximate to SCI severity (Strickland et al., 2011). Additional support for a severity effect on transcription comes from mRNA data. De Biase et al. (2005) observed strong differences in gene expression in contusive injuries of different severities, with a dramatic increase in the number of genes with altered expression in moderate injuries relative to mild and severe ones. Thus, injury severity could be an important and interesting source of variation of microRNA expression that would merit further studies. Other injury features contribute to condition the microRNA profile of the spinal cord. Recently, Ziu et al. (2013) have compared the expression of six microRNAs after SCI with variable compression times showing that the duration of compression significantly alter the expression of different microRNAs.

**Table 1:** Global analyses of microRNA expression after SCI.

Study	SCI model	miRNA expression changes
Liu et al. (2009)	Animal: Sprague Dawley rats	4 hpo: 18 down, 23 up
	Contusion, NYU impactor (10 g, 12.5 mm)	1 dpo: 30 down, 27 up
	Level: T10	7 dpo: 30 down, 30 up
	Sampling: 4 h, 1 and 7 days	
Nakanishi et al. (2010)	Animal: C57BL/6 mice	12 hpo: 5 down, 5 up
	Compression, forceps (10 s).	
	Level: T11–T12	
	Sampling: 12 h	
Strickland et al. (2011)	Animal: Sprague Dawley rats	4 and 14 dpo: 32 down, 4 up
	Contusion, MASCIS impactor (10 g, 12.5 mm)	
	Level: T12–T13	
	Sampling: 4 and 14 days	
Jee et al. (2012a,b), Im et al. (2012)	Animal: ICR mice	265 exp. changes
	Transection	
	Level: T9–T10	
	Sampling: ?	
Yunta et al. (2012)	Animal: Wistar rats	1 dpo: 0 down, 0 up
	IH spinal cord impactor (200 kdynes)	3 dpo: 46 down, 5 up
	Level: T8	7 dpo: 192 down, 11 up
	Sampling: 1, 3, and 7 days	
Hu et al. (2013b)	Animal: Sprague Dawley rats	1 dpo: 5 down, 9 up
	Contusion, mod. Allen Weight Drop (8 g, 40 mm)	3 dpo: 5 down, 3 up
	Level: T10	
	Sampling: 1 and 3 days	
Hu et al. (2013a)	Animals: Rats	2 dpo: 10 down, 38 up
	Ischemic-Reperfusion	
	Level: lumbo-sacral segments	
	Sampling: 2 days

*Summary of models and results employed in published microarray analyses of miRNA expression in spinal cord injury. In all cases, data from injured animals was compared to data from Sham injured animals. Information from several studies was very limited and we would encourage authors to submit microarray data to public repositories as part of the process of publication, as suggested by the Microarray Gene Expression Data Society (http://www.mged.org). T, thoracic level; hpo, hours post-operation; dpo, days post-operation; up and down indicate up- and down-regulated microRNAs.*

Besides general variability in the microRNA expression patterns, Strickland et al. (2011) and our group (Yunta et al., 2012) observed a global downregulation of miRNA expression after a moderate contusive injury. Interestingly, studies by De Biase et al. (2005) and Byrnes et al. (2006) showed that moderate contusive injuries present a significant bias toward gene upregulation with a maximum at 7 days after injury, that contrast with the more balanced numbers of up- and downregulated genes observed in severe and mild injuries. Thus, the increase in the number of upregulated mRNA correlates with the general decrease of miRNA expression, which becomes particularly evident 1 week after injury (Yunta et al., 2012). Bioinformatic analyses based on miRNA target prediction data allowed us to show that almost one-third of the upregulated mRNAs in De Biase et al.’s (2005) study where targets of the downregulated microRNAs (Yunta et al., 2012). It is tempting to hypothesize that a generalized decrease in miRNA abundance reduces post-transcriptional regulation and thus causes an increase in mRNA levels after moderate SCI. Two questions immediately arise: why in moderate injuries? and, how do injuries regulate microRNA expression or biogenesis? Although we have no information to approach the first question, some is available to explore the effects of injuries on microRNA biogenesis. MicroRNA biogenesis can be regulated at different stages. The first layer governing miRNA abundance is the regulation of pri-miRNA transcription by binding of transcription factors (Treiber et al., 2012). C-Myc is one of such transcription factors, which is known to directly upregulate miR-17-92 cluster and, at the same time, to cause a widespread repression of microRNA expression (Chang et al., 2008). Interestingly, this transcription factor is significantly overexpressed at 4 and 24 h following a SCI (Di Giovanni et al., 2003). In agreement, the levels of several microRNAs repressed by c-Myc in Chang et al. (2008) experiments – miR-26a, miR-26b, miR-29a-c, miR-34a, miR-146a, miR-30a-e, let-7a, let-7d, let-7g, miR-99b, and miR-125 – become significantly decreased after SCI in our analysis whereas two members of the miR-17-92 cluster (miR-20a and miR-17) appear upregulated (Yunta et al., 2012). In addition to transcriptional regulation, processing of the pri- and pre-miRNA transcripts can be also regulated (Treiber et al., 2012). Blockage or downregulation of key proteins in the biogenesis pathway such Dicer, Drosha, DGCR8, or Exportin 5 leads to a reduction in the abundance of mature microRNAs and a accumulation of pri- and pre-microRNAs (Lee et al., 2008). Examples of this regulatory pathway are common in cancer (Lu et al., 2005) but also in other processes, such as liver regeneration after damage (Shu et al., 2011). Little is known about the variation in the expression and function of the microRNA biogenic machinery after SCI, however, Jee et al. (2012a) have recently described a downregulation of Dicer 7 days after contusive spinal cord. Dicer downregulation is consistent with the observed general depletion in miRNA abundance observed by Strickland et al. (2011) and Yunta et al. (2012). Additionally, growth factors such as bone morphogenetic proteins (BMPs) and TGF-β – overexpressed after SCI (McTigue et al., 2000; Xiao et al., 2010) – can contribute to regulate the microRNA biogenesis through activation of Smad proteins, which bind to pri-miRNA and enhance their Drosha-mediated processing to pre-miRNA (Davis et al., 2010). To what extent all these mechanisms contribute to the different patters of microRNA expression following SCI remains to be elucidated.

Microarray data allows identifying consistent changes in microRNA abundance following SCI. Considering those changes observed in at least two different studies, 36 microRNAs show consistent patterns of change (see **Table 2**). These changes illustrate the complexity of interpreting the microRNA expression changes from microarray data in the SCI due to the cellular heterogeneity of the spinal cord and the multiple changes that take place after injury. Expression changes in heterogeneous samples such as the spinal cord actually correspond to the weighted mean of the transcription programs of all cell types present in the sample (Lu et al., 2003). Thus, the observed expression changes may result either from changes in gene expression within a given cell type or to changes in the relative abundance of the expressing cell types, which severely constraints the conclusion that can be derived from the expression data (Wang et al., 2006; Gosink et al., 2007). In fact, heterogeneity could be a major reason why many gene expression analyses fail a rigorous validation (Clarke et al., 2010). After injury, the spinal cord experiences changes in the relative proportions of different cell types due to the necrotic and apoptotic death of neurons and oligodendrocytes (Grossman et al., 2001; Rowland et al., 2008) and the infiltration of immune cells (Grossman et al., 2001; Profyris et al., 2004). The death of specific cell types explains the downregulation of microRNAs associated with neurons, such as miR-124 and oligodendrocytes, miR-219 (Smirnova et al., 2005; Lau et al., 2008). Both microRNA show a sustained decrease in their levels that follows the progression of cell death in these neural cells. Interestingly, Liu et al. (2009) observed an increase in miR-124 abundance during the first 4 h. A similar upregulation was previously described in brain ischemia-reperfusion by Jeyaseelan et al. (2008), which proposed that such increase “indicates that injured brain cells could be actively involved in regeneration during the first 24 h of reperfusion.” In parallel, the infiltration of immune and vascular cells explains the overexpression of specific microRNAs. The best characterized is miR-223, a neutrophil microRNA (Lindsay, 2008; Sonkoly and Pivarcsi, 2009; Izumi et al., 2011), whose upregulation reflects the infiltration of these immune cells during the acute phase of the spinal cord (Izumi et al., 2011; Yunta et al., 2012). Similarly, miR-451 – a red blood cell marker (Merkerova et al., 2008) – appears clearly increased in the first hours after injury to be later repressed or returned to control condition probably reflects the entrance of erythrocytes in the damaged area during the acute phase and their clearance later on.

**Table 2:** Prominent changes in microRNA expression in murine models of spinal cord injury.

	Time after injury						
MicroRNA	4 h	12 h	1 days	3 days	4 days	7 days	14 days
miR-1		Up ^2^	Up ^1^	Down ^4^	Down ^3^	Up ^1^/Down ^4^	Down ^3^
miR-100	Up ^1^		Down ^1^			Down ^1,4^	
miR-103	Up ^1^		Down ^1^			Down ^1,4^	
miR-107	Up ^1^		Down ^1^			Down ^1,4^	
miR-124	Up ^1^	Down ^2^	Down ^1^	Down ^4^	Down ^3^	Down ^1,4^	Down ^3^
miR-125b-3p			Down ^5^			Down ^4^	
miR-126			Down ^5^	Down ^5^		Down ^4^	
miR-127	Up ^1^		Down ^1^	Down ^4^		Down ^1,4^	
miR-128	Up ^1^		Down ^1^	Down ^4^		Down ^1,4^	
miR-129-1					Down ^3^		Down ^3^
miR-129-2					Down ^3^		Down ^3^
miR-129-3p		Down ^2^				Down ^4^	
miR-129*	Down ^1^		Down ^1^			Down ^1,4^	
miR-133a	Up ^1^	Up ^2^	Down ^1^	Down ^4^		Down ^1,4^	
miR-133b	Up ^1^	Up ^2^	Down ^1^	Down ^4^		Down ^1,4^	
miR-138	Down ^1^		Down ^1^	Down ^4^		Down ^1,4^	
miR-146a			Up ^1^			Up ^1,4^	
miR-17			Up ^1^	Up ^4^		Up ^1^	
miR-181a	Up ^1^		Down ^1^			Down ^1,4^	
miR-21			Up ^1^	Up ^4,5^	Up ^3^	Up ^1,4^	Down ^3^
miR-219-2-3p	Down ^1^		Down ^1^			Down ^1,4^	
miR-219-5p	Down ^1^		Down ^1^			Down ^1,4^	
miR-223	Up ^1^	Up ^2^	Up ^1,4^	Up ^4^	Up ^3^	Up ^1,4^	Up ^3^
miR-30b-5p	Down ^1^		Down ^1^			Down ^1,4^	
miR-30c	Down ^1^		Down ^1^			Down ^1,4^	
miR-30d	Down ^1^		Down ^1^			Down ^1,4^	
miR-338*	Down ^1^		Down ^1^			Down ^1,4^	
miR-342		Down ^2^				Down ^4^	
miR-34a	Down ^1^		Down ^1^	Down ^4^		Down ^1,4^	
miR-379*	Down ^1^		Down ^1^	Down ^4^		Down ^1,4^	
miR-451	Up ^1^	Up ^2^	Down ^1^/Up vs 7 dpo ^4^			Down ^1^	
miR-487b	Up ^1^		Down ^1^			Down ^1,4^	
miR-495	Down ^1^	Down ^2^	Down ^1^	Down ^4^		Down ^1,4^	
miR-708			Down ^5^	Down ^4^		Down ^4^	
miR-99a	Up ^1^		Down ^1^	Down ^5^		Down ^1,4^	
miR-let-7b			Down ^5^	Down ^5^		Down ^4^	

*The table details the changes in microRNA expression following spinal cord injury of dysregulated microRNAs according to, at least, two different studies. Data derived from the following microarray analyses: ^1^Liu et al. (2009); ^2^Nakanishi et al. (2010); ^3^Strickland et al. (2011); ^4^Yunta et al. (2012); ^5^Hu et al. (2013a),2013b. Unless stated, Up and Down correspond to upregulated and downregulated respect to Sham control individuals respectively.*

## MicroRNAs IN THE REGULATION OF INFLAMMATION FOLLOWING SCI

**FIGURE 1:**
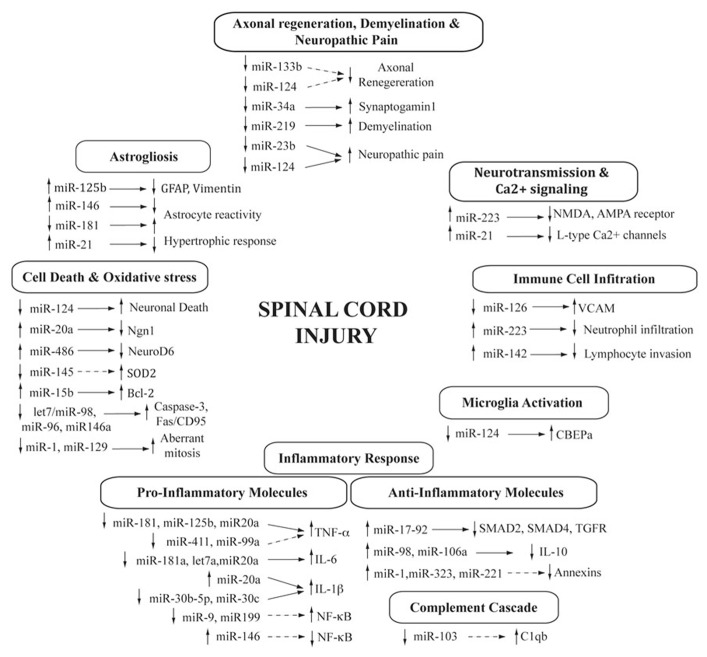
**MicroRNAs in the spinal cord injury pathophysiology.** Diagram showing the cascade of processes triggered after SCI and the contribution of differentially expressed microRNAs. Changes in microRNA expression is indicated either as increased (up arrow) or decreased (down arrow) after SCI. Target genes are also shown (confirmed, direct regulation are indicated with a solid arrow, whereas those indirect or predicted are represented with a dashed arrow).

Spinal cord injury activates an inflammatory response that is initiated by the alteration of the blood–spinal cord barrier, followed by the sequential infiltration of peripheral immune cells, the activation of the microglia and the induction of inflammatory signaling pathways. MicroRNAs play important roles in controlling signaling pathways and the dynamics of the immune response during pathogenic immunological conditions (Lindsay, 2008; Sonkoly and Pivarcsi, 2009; Chen et al., 2013). Following SCI, several microRNAs undergo expression changes that can be related to the immune response, either associated with the invading immune cells or participating in the modulation of inflammatory pathways (**Figure 1**).

Immediately after the injury, the blood–spinal cord barrier becomes disturbed and the blood immune cells infiltrate the damaged area (Profyris et al., 2004; Jones et al., 2005). Early increases in the expression of CAMs play a key role in the process of immune cells recruitment and extravasation into the nervous tissue. Some of these expression changes can be controlled by microRNAs. In particular, the upregulation of *VCAM1* mRNA (Aimone et al., 2004) occurs in parallel to the downregulation of its regulator miR-126 (Harris et al., 2008) during the first week after injury (Yunta et al., 2012; Hu et al., 2013b). The subsequent immune cell infiltration is responsible for several changes in the microRNA profile of the spinal cord, as previously commented. Neutrophil infiltration explains the upregulation of miR-223 (Lindsay, 2008; Sonkoly and Pivarcsi, 2009; Izumi et al., 2011), whereas increased expression of the lymphocyte specific miR-142 (Wu et al., 2007) correlates with the access of these immune cells to the injury site during the first week (Yunta et al., 2012). MicroRNAs are also involved in the activation of microglia and macrophages. Particularly, the downregulation of miR-124 contributes to resting phenotype of microglia by targeting CEBPα, a master transcription factor important for the development of myeloid cells (Ponomarev et al., 2011; Guedes et al., 2013). miR-124 shows a sustained downregulation after injury (Deo et al., 2006; Liu et al., 2009; Nakanishi et al., 2010; Yunta et al., 2012) that may underlie microglial activation. However, miR-124 is a well-characterized neuronal microRNA and its downregulation likely reflects the extension of neuronal death that characterizes the secondary damage of SCI.

The inflammatory response is modulated by a number of key molecular immune mediators, such as cytokines, chemokines (TNF-α, IL-6, IL-1β) or the complement cascade (C1qb; Hausmann, 2003; Profyris et al., 2004; Jones et al., 2005), which are known targets of microRNAs. Particularly, overexpression of three key pro-inflammatory cytokines that modulate the inflammatory response in the SCI is likely regulated by microRNA experiencing expression changes after injury. Different SCI studies (Liu et al., 2009; Yunta et al., 2012) have suggested that increasing levels of the pro-inflammatory and pro-apoptotic factor TNF-α after injury (Tyor et al., 2002) may result from downregulation of its regulators miR-181 (Hutchison et al., 2013) and miR-125b (Tili et al., 2007) after SCI (Liu et al., 2009; Yunta et al., 2012; Hu et al., 2013b). Other microRNAs, such as miR-411, miR-99a, that appear downregulated after SCI (Liu et al., 2009; Yunta et al., 2012; Hu et al., 2013b) have been also predicted to target TNF-α by bioinformatics analysis. On the other hand, the increased levels of cytokine IL-6 during the first days after injury correlate with a reduced expression of its regulators let-7a (Iliopoulos et al., 2009) or miR-181a (Tili et al., 2007; Iliopoulos et al., 2009). Finally, the observed downregulation of miR-30b-5p and miR-30c during the first week after injury (Liu et al., 2009; Yunta et al., 2012) could be related to the overexpression of their target IL-1β. Additionally, it has been recently described that upregulation of miR-20a induces a cytotoxic environment with increased IL-6, TNF-α, IL-1-β, and COX-2 expression in the spinal cord (Jee et al., 2012b) due to miR-20a-mediated inhibition of neurogenin 1 (*NGN-1*). Inhibition of this microRNA – which is upregulated after injury (Liu et al., 2009; Jee et al., 2012b) – lead to a significant improvement in functional recovery associated to a reduced inflammation and the increased survival of motor neurons (Jee et al., 2012b).

Interestingly, pro-inflammatory cytokines lead to the activation of the NF-κB signaling pathway, which is also under microRNAs regulation (Ma et al., 2010). In particular, downregulation of miR-9 and miR-199 (Yunta et al., 2012) may induce the overexpression of the NF-κB pathway genes *p50NFkB* and *ikkb* (Chen et al., 2008; Bazzoni et al., 2009; Wang et al., 2011). The increased expression of miR-21 may also contribute to the regulation of this pathway but its role is less clear, as it exhibits both pro- and anti-inflammatory effects. miR-21 targets PTEN, a negative regulator of NF-κB (Iliopoulos et al., 2010), but also PDCD4, which promotes NF-κB activation and inhibits the expression of IL-10 (Frankel et al., 2008; Sheedy et al., 2010; Young et al., 2010). On the opposite side, expression changes in several microRNAs that have been observed after SCI may attenuate the activation of NF-κB pathway, contributing to the attempts of the damaged spinal cord to recover homeostasis (Bareyre and Schwab, 2003). These changes include the increased expression of miR-146a at 7 days after injury (Liu et al., 2009; Yunta et al., 2012), which negatively regulates NF-κB expression (Taganov et al., 2006; Ma et al., 2010). Interestingly, miR-146a expression is induced by NF-κB and, thus, its overexpression at 7 days after injury may be consequence of the increased levels of NF-κB in the previous days (Bethea et al., 1998), forming a negative feedback that may cause the inactivation of NF-κB pathway.

A second group of factors with a prominent role in inflammation after SCI are the complement proteins (Brennan et al., 2012). Complement activation is involved in the removal of cellular debris, but it may also promote clearance of mildly damaged cells contributing to secondary cell death and demyelination. Complement protein C1qb increases its expression in the first day after injury and persist upregulated at least 5 weeks later (Aimone et al., 2004). C1q knockout mice show improved locomotor recovery and reduced secondary tissue damage after contusive SCI (Galvan et al., 2008). Interestingly, C1qb is a predicted target of miR-103 (Perri et al., 2012), which appears downregulated in the first week after injury (Liu et al., 2009; Yunta et al., 2012). Thus, miR-103 downregulation could be responsible for the overexpression of the complement protein C1qb and its associated deleterious effects.

Inflammation is also stimulated through the inhibition of anti-inflammatory pathways, such as the downregulation of pSMAD2, SMAD4, and TGFBR2 by observed upregulation of members of the miR-17-92 microRNA cluster (Mestdagh et al., 2010) or the silencing of the anti-inflammatory neuroprotective cytokine IL-10 by miR-98, miR-106a (Sharma et al., 2009; Liu et al., 2011). Many other microRNAs have been related to inflammation in SCI based on *in silico* predictions. Bioinformatics analyses predict that anti-inflammatory mRNAs annexin A1, annexin A2 and annexin A7 mRNAs are potential targets of the SCI, upregulated microRNAs miR-221, miR-1, and miR-323, respectively (Liu et al., 2009; Hu et al., 2013a). The list also includes miR-127, miR-411, and miR-34a, significantly downregulated after SCI in adult rats and which according to Liu et al. (2009) should lead to increased inflammation. Other pro-inflammatory microRNAs, such as miR-152, miR-214, miR-206, and miR-221, show significant changes in isolated studies or even show opposite expression trend (i.e., miR-1) in different studies (Liu et al., 2009; Strickland et al., 2011; Yunta et al., 2012). All these results will require further assays to elucidate the role in the inflammatory process of the SCI.

## MicroRNA REGULATION OF CELL DEATH REGULATION IN SCI

Cell death is a hallmark of the pathophysiology of SCI (Crowe et al., 1997; Liu et al., 1997). The programed cell death (apoptosis) that characterizes the SCI secondary damage is a gene-controlled process that is stimulated or inhibited by a variety of regulatory factors including several microRNAs (Wang, 2010). Previous studies have shown that SCI alters the transcription levels of a substantial number of genes associated with the regulation of apoptosis (Aimone et al., 2004). The resulting scenario is complex, combining both temporal and spatial changes in the expression of both pro and anti-apoptotic signals. In agreement, many microRNAs have been proposed to promote or inhibit cell death during the course of SCI, with discrepancies among microRNA expression profiling studies. These discrepancies highlight the intricate roles that miRNAs play in the regulation of cellular processes and the difficulty to identify the precise activity of dysregulated microRNAs. However, two recent studies have provided direct evidence of microRNAs involvement in cell death modulation following SCI (**Figure 1**).

The first study deals with miR-20a, which shows an increased expression early after injury (24 h) that persists at least for 1 week (Liu et al., 2009; Jee et al., 2012b). miR-20a inhibits the expression of Ngn1, a protein with a key role in maintenance of cell survival, self-renewal, and neurogenesis in normal and injured spinal cords. Silencing Ngn1 by siNgn1 or infusion of miR-20a into uninjured mouse spinal cord reproduced SCI-like symptoms including apoptotic death of neural cells, whereas the administration of anti-miR-20a decreased apoptosis, reduced tissue damage and functional deficits were significantly ameliorated (Liu et al., 2009; Jee et al., 2012b). In a similar way, miR-486 is upregulated at 7 days after injury (Jee et al., 2012a). miR-486 represses neurogenic differentiation 6 (NeuroD6), a protein that promotes neuronal survival by increased expression of the ROS scavenger proteins (Jee et al., 2012a). Infusion of miR-486 into the normal spinal cord of mice reproduces SCI symptoms including increased neuronal death, whereas silencing miR-486 after SCI produced a decrease in the magnitude of neuronal death and led to a significant improvement in motor recovery. Therefore upregulation of miR-486 following SCI promotes neurodegeneration by suppressing NeuroD6, pointing to this microRNA as a potential target for therapeutic interventions (Jee et al., 2012a).

Further evidences of the roles of microRNAs on secondary cell death come from microRNA expression changes that are accompanied by changes in the expression of their apoptotic gene targets. Several pro and anti-apoptotic microRNAs act on key apoptosis molecules, such as caspases, Fas/CD95, c-Myc, TNF-α, or members of the BCL-2 family. For example, the decreased expression of the let-7/miR-98 family members miR-96 and miR-146a (Liu et al., 2009; Strickland et al., 2011; Yunta et al., 2012) would promote apoptosis by increasing the expression of their targets, the pro-apoptotic proteins caspase 3 (Citron et al., 2000; Aimone et al., 2004) and Fas/CD95 (Casha et al., 2001). On the contrary, overexpression of miR-21 would protect neural cells from death by repressing the expression of the pro-apoptotic molecules Fas ligand (Buller et al., 2010), TPM1 and PTEN (Hafez et al., 2012; Han et al., 2012), and PDCD4 (Frankel et al., 2008). BCL-2 modulation is highly representative of the complexity of microRNA regulation of cell death in SCI. Upregulation of miR-15b (Liu et al., 2009) would decrease BCL-2 (Cimmino et al., 2005; Saito et al., 2006) and induce apoptosis. However, upregulation of miR-15b is counteracted by the decreased expression during the first week of miR-138 and miR-148b, which also target BCL-2 (Liu et al., 2009; Yunta et al., 2012). Downregulation of these microRNAs is broadly consistent with the increase in the number of BCL-2-positive cells present 3 days after injury (Saito et al., 2000; however, see Qiu et al., 2001), although microRNA downregulation extends throughout the 7-day period after injury, which is the time-point when the number of BCL-2-positive cells is progressively reduced. Other microRNAs targeting BCL-2 appear dysregulated after SCI. Regulation of BCL-2 by miR-107 was discussed in the profiling study by Liu et al. (2009). These authors observed a miR-107 upregulation 4 h after injury, which they proposed should decrease BCL-2 levels and induce apoptosis, to be later downregulated at 7 dpi (also observed in Yunta et al., 2012) promoting cell survival. miR-1 represents a puzzling case that appears upregulated in Liu et al. (2009) and downregulated in the analyses by Strickland et al. (2011) and Yunta et al. (2012).

In addition to modulation of genes that regulate apoptosis, microRNAs also participate in the disruption of the calcium signaling or the oxidative stress events triggered after SCI that contribute to secondary cell death. Expression of the gene coding for the Ca^2^^+^-related genes such as Ca^2^^+^ pump, voltage-gated (L-type) Ca^2^^+^ channels or Ca^2^^+^-permeable ionotropic glutamate (AMPA) channels, is decreased with injury and post-transcriptionally regulated by microRNAs. Several studies have shown that upregulated miR-223 reduces the expression of the NR2B and GluR2 subunits of the NMDA and AMPA receptors, respectively (Kaur et al., 2012). Similarly, decreased expression of voltage-gated (L-type) Ca^2^^+^ channels may be result of upregulated miR-21 (Carrillo et al., 2011). These could cause an increment of intracellular Ca^2^^+^ concentration level that accompanies traumatic SCI, and could trigger mechanisms of secondary cell death, such as calpain activation. MicroRNAs also play an important role in the regulation of oxidative stress, a hallmark of the secondary damage of SCI that has received much attention in the attempts to develop effective therapies (Jia et al., 2012). Recent reports have demonstrated that miR-486 repress the expression of NeuroD6, a neuroprotective protein that promotes the expression of ROS scavenger proteins, such as GPX3, selenoprotein-N, and thioredoxin (Jee et al., 2012a). Upregulation of miR-486 – observed in motor neurons at 7 days after injury in murine models of SCI – leads to the repression of NeuroD6 expression, and consequently to a decrease in the expression of ROS scavenger proteins and increased neurodegeneration mediated by oxidative stress (Jee et al., 2012a).

Microarray analyses revealed increased expression of genes associated with anti-oxidant actions, such as SOD1, SOD2, catalase, and GPX (Di Giovanni et al., 2003; Aimone et al., 2004). This overexpression of the mitochondrial SOD2 gene (*sod2*) 7 days after injury (Santoscoy et al., 2002; Sugawara et al., 2002) is consistent with the downregulation of its modulator miR-145 (Dharap et al., 2009) described in Yunta et al. (2012). However, the bioinformatics analysis performed by Liu et al. (2009) revealed that some anti-oxidant genes such as SOD1 and catalase gene are potential targets of the upregulated miR-206, miR-152, and miR-214. Moreover, it has been proposed that downregulation of miR-1 and miR-129, which regulate transcription, differentiation or prevent post-mitotic cells from re-entering the cell cycle, could cause neural cells to become aberrantly mitotic, increasing the number of apoptotic cells observed at the injury site after SCI (Bhalala et al., 2013).

## MicroRNA MODULATION OF ASTROCYTE REACTIVITY AND GLIAL SCAR

Astrogliosis is another hallmarks of the cellular response to SCI. It consists in an early hypertrophic neuroprotective phase followed by a hyperplasic phase characterized by the formation of a dense glial scar that inhibits CNS regeneration during the subacute and chronic phases of the SCI (Sofroniew, 2009). Recent genomic analyses have shown reactive astrogliosis is associated to a rapid, but quickly attenuated, induction of gene expression (Zamanian et al., 2012). Increasing evidence supports the involvement of several microRNAs in the regulation of the astrocyte response to injury, including four microRNAs that appear dysregulated in studies of SCI. The best characterized is miR-21. Its expression increases in a time-dependent manner following SCI (Liu et al., 2009; Bhalala et al., 2012; Yunta et al., 2012; Hu et al., 2013b) and is highly expressed in astrocytes during the chronic stage (Bhalala et al., 2012). miR-21 expression after SCI shows a marked spatial pattern, with highest expression in the astrocytes adjacent to the lesion area (Bhalala et al., 2012). The role of miR-21 in astrogliosis has been studied in detail using transgenic mice that overexpress in astrocytes either miR-21 or a miRNA sponge designed to inhibit miR-21 function (Bhalala et al., 2012). The results from these studies demonstrate that miR-21 overexpression in astrocytes abrogates the hypertrophic astrocytic response after severe SCI, which is consistent with previous studies *in vitro* (Sahni et al., 2010; Sayed and Abdellatif, 2011). On the contrary, miR-21 inhibition enhances the hypertrophic response in early and chronic stages after SCI (Bhalala et al., 2012). BMP signaling following SCI mediates the miR-21 and astrocytic response through the opposing effects of the BMP receptors BMPR1a and BMPR1b (Sahni et al., 2010). BMPR1a signaling decreases levels of miR-21 and induces reactive astrocytic hypertrophy, whereas BMPR1b signaling increases miR-21 levels and negatively regulates astrogliosis. These findings point to the BMP–BMPR–miR-21 axis as a key regulator of astrocytic hypertrophy and glial scar progression after SCI, modulating the pro-reactive effects of the inflammatory signaling.

A second microRNA that has been related to astrogliosis is miR-125b. Overexpression of miR-125b correlates with the overexpression of the astrogliosis markers GFAP and vimentin in several neurological disorders (Pogue et al., 2010). *In vitro* studies show that miR-125b downregulation in IL-6 stimulated reactive astrocytes increases the expression of its target cyclin-dependent kinase inhibitor 2A (CDKN2A), a negative regulator cell growth, and attenuates cell proliferation. Thus, evidences indicate that miRNA-125b upregulation contributes to astrogliosis. However, contrary to expectations, miR-125b appears downregulated during the first week after injury (Yunta et al., 2012), which would contribute to inhibit astrocyte proliferation and astrogliosis.

The miR-181 family of miRNAs is another candidate for post-transcriptional regulation of neuroinflammation and reactive gliosis (Hutchison et al., 2013). miR-181s are constitutively expressed in astrocytes but inflammation causes its downregulation, in agreement with *in vivo* observations following SCI (Liu et al., 2009; Yunta et al., 2012). Genetic studies demonstrated that miR-181 inhibits the production of multiple pro-inflammatory cytokines (TNF-α, IL-6, IL-1β, IL-8, LIF, and HMGB1) and increases the levels of the anti-inflammatory cytokine IL-10 (Hutchison et al., 2013) in cultured astrocytes under LPS inflammatory exposure. Thus, miR-181s act as negative regulators of astrogliosis, reducing the expression of reactivity promoters such as pro-inflammatory cytokines (Balasingam et al., 1994; Sofroniew, 2009) and FGF2 (Goddard et al., 2002), and increasing the expression of reactivity inhibitors, such as IL-10 (Balasingam and Yong, 1996).

Similarly to miR-181s, miR-146a is a negative regulator of the astrocyte response to inflammation and, consequently, a negative regulator of astrogliosis (Iyer et al., 2012). miR-146a is expressed in reactive astrocytes in the areas of prominent gliosis (Iyer et al., 2012), and appears upregulated 7 dpi after SCI (Liu et al., 2009; Strickland et al., 2011; Yunta et al., 2012) as well as in several neurodegenerative pathologies (Junker et al., 2009; Cui et al., 2010; Iyer et al., 2012). Studies with astroglioma cell lines and primary astrocytes have shown that IL-1β stimulation induces a prominent upregulation of miR-146a expression. Overexpression of miR-146a has anti-inflammatory effects and significantly reduces the expression of signaling molecules downstream IL-1β, such as IRAK-1, IRAK-2, and TRAF-6 and inhibits the release of pro-reactive and pro-inflammatory factors, including IL-6 and COX-2 (Iyer et al., 2012). Thus, miR-146a forms a negative feedback of the IL-1β signaling, being induced by IL-1β but blocking the expression of its downstream response. Overexpression of miR-146a following SCI adds to the overexpression of miR-21 and miR-125b to limit astrocyte reactivity. On the contrary, downregulation of miR-181 family members would promote astrocyte reactivity, indicating a fairly complex regulation of the astrocyte response. It is interesting to note that according to recent studies, the disruption of microRNA biogenesis by deletion of Dicer leads to an altered mature astroglial transcriptome signature that resembles to a reactive state (Tao et al., 2011), which adds a further layer of complexity to the whole picture.

## THE ROLES OF microRNAs IN AXONAL REGENERATION, MYELINATION, AND OTHER PROCESSES

Although less studied, microRNAs also seem to contribute to the regulation of other pivotal processes in the SCI pathophysiology, such as axonal regeneration, remyelination, or pain. As we mentioned before, failure to produce a sustained regenerative response is one of the critical features of the CNS. Local environmental clues are largely responsible for the lack of regeneration but intracellular specific features associated to neural cell maturation are also involved (see, for example, Goldberg, 2003). Different evidences indicate that microRNAs can contribute to these changes. In fact, microRNAs have a critical role in neurite outgrowth in post-mitotic neurons as demonstrated in Dicer conditional knockout mice. In this animals, silencing of Dicer and the subsequent inhibition of the microRNA biogenesis causes defects in neurite outgrowth and decreased soma size but has not influence in neurogenesis, cortical patterning or cell survival (Hong et al., 2013). Additional evidences have been obtained from the zebrafish, a model of spontaneous axonal regeneration in the damaged spinal cord (Becker et al., 1997). In this fish, miR-133b is overexpressed in regenerating neurons following SCI and its inhibition using antisense morpholinos result in reduced axonal regeneration (Yu et al., 2011). Functionally, miR-133b contributes to spinal cord regeneration through the downregulation of its target RhoA, a small GTPase that inhibits axonal growth. Contrary to zebrafish, miR-133b shows a significant downregulation at 1 and 7 days after contusive SCI in mammals (Liu et al., 2009; Yunta et al., 2012), which may contribute to their reduced neuroregenerative capacity. miRNA-124 presents a somehow similar behavior that may also contribute to reduce axonal regeneration after SCI. Previous studies have shown that miR-124 overexpression in differentiating mouse P19 cells promotes neurite outgrowth, while miR-124 inhibition reduces it (Yu et al., 2008). Thus the observed miR-124 downregulation after SCI may also contribute to hinder the axonal regenerative capacities of spinal cord neurons. However, microRNAs may also contribute to the activation of pro-regenerative gene programs after injury. Di Giovanni et al. (2005a) described the overexpression at 7 and 28 days after SCI of a gene cluster that comprise known promoters of the neural plasticity and the neurite outgrowth, including synaptotagmin-1. Interestingly, synaptotagmin-1 is a target of miR-34a (Agostini et al., 2011), and its upregulation after SCI is consistent with the observed downregulation of miR-34a at 3 and 7 after injury (Liu et al., 2009; Yunta et al., 2012).

The progressive loss of myelin in the areas surrounding the injury is another critical feature of the SCI that results from the combined effects of damage to oligodendrocytes and remyelination failure. Evidences have confirmed that microRNA loss of function due to Dicer1 ablation in mature oligodendrocytes causes demyelination, gliosis, and neuronal degeneration (Shin et al., 2009; Dugas et al., 2010). More precisely, Shin et al. (2009) identified miR-219 as a central actor in myelin maintenance and remyelination. miR-219 is highly expressed in mature oligodendrocytes and when is lost due to Dicer1 ablation, miR-219 target ELOVL7 increases its expression resulting in lipid accumulation in myelin-rich areas and disrupting the stability of the membranes (Shin et al., 2009). Strikingly, miR-219 abundance is markedly reduced after SCI (Liu et al., 2009; Yunta et al., 2012) although this decrease may also reflect the loss of spinal cord oligodendrocytes that takes place after injury. Further studies are needed to determine the contribution of microRNAs in demyelination and remyelination and to evaluate their use as therapeutic tools in the SCI and other CNS pathologies.

In addition to their direct roles in most processes implicated in the pathophysiology of the SCI, microRNAs are also involved in the functional consequences of SCI, including the neuropathic pain. Neuropathic pain is the manifestation of maladaptive plasticity in the nervous system characterized by pain in the absence of a stimulus and reduced nociceptive thresholds (Scholz and Woolf, 2007). It is a debilitating accompaniment of SCI that affects up to 50% SCI patients and limit their ability to achieve an optimal level of activity (Mann et al., 2013). Plastic changes in sensory neuron excitability are considered the cellular basis of neuropathic pain, although a growing body of evidence also implicates activated microglia and astrocytes as key players in the development of pain (Scholz and Woolf, 2007). Although information on the roles of miRNAs in neuropathic pain following SCI is very restricted, available evidences indicate that microRNAs expression at the spinal cord respond to pain induction (Kusuda et al., 2011; Genda et al., 2013; Li et al., 2013), although evidences are sometimes contradictory (Brandenburger et al., 2012). There are also evidences of the contribution of microRNAs in the development of central neuropathic pain. Recently, Im et al. (2012) demonstrated that SCI reduced the expression of miR-23b in GABAergic neurons from the spinal cord while increasing the levels of NADPH oxidase 4 (NOX4), a target of miR-23b and a key factor in the production of ROS and pain induction. Reduction of NOX4 expression and neuropathic pain was observed after infusion of miR-23b to the spinal cord confirming the involvement of this microRNA in pain regulation. Similarly, intrathecal administration of miR-124 inhibits the activation of spinal cord microglia, reducing inflammation and preventing neuropathic pain (Willemen et al., 2012). Many other microRNAs have been described to affect neuropathic pain at different levels, particularly at the dorsal root ganglia. However, a review of this complex system lies beyond the scope of the present article.

## CLINICAL APPLICATIONS: microRNA-BASED THERAPEUTIC AND DIAGNOSTIC TOOLS

The capability of individual microRNAs to reduce the expression of numerous components of cellular networks supposes an opportunity to modulate the cell phenotypes by manipulating the expression or function of microRNAs. The possibility of a therapeutic use of the miRNAs is highly appealing due to their capability to modulate entire gene programs though tuning, not blunting, the expression of their targets (Hydbring and Badalian-Very, 2013). Moreover, microRNA deregulation has a critical role in a wide range of pathologies, and, indeed, several studies have identified specific deregulated microRNAs that can constitute potential targets of therapeutic approaches in a wide range of pathologies. As a consequence, in the short time from its discovery in humans, a microRNA-based therapeutic for suppression of hepatitis C virus has already entered phase II clinical trial and several others are on their way (see review in Hydbring and Badalian-Very, 2013).

Therapeutic approaches are based on local or systemic administration of either antagonists (anti-miRs) of endogenous microRNAs that show a gain-of-function in diseased tissues or mimics that replace downregulated microRNAs. Both modulators incorporate chemical modifications (phosphorothionate backbones, locked nucleic acids or LNAs, etc.) to confer resistance to nucleases, increase stability during delivery, and facilitate cellular uptake. Anti-miRs are siRNAs designed to inhibit miRNAs through complementary base pairing, in a similar way to siRNA. Since binding is irreversible, the resulting miRNA duplex cannot be processed by RISC and/or degraded. Anti-miRs are synthesized as short single-stranded oligonucleotides of small size that make delivery possible without vehicle-systems. On the contrary, administration of microRNA mimics, also known as “miRNA replacement therapy,” aims to re-introduce miRNAs into diseased cells that are normally expressed in healthy cells. miRNA mimics is expected to re-activate pathways required for normal cellular welfare and block those driving disease. To be processed correctly by the cellular RNAi-machinery, miRNA mimics need to be double-stranded, which confer them greater chemical complexity and larger size. Thus, miRNA mimics require delivery techniques similar to those employed in siRNA therapeutics, including microvesicles, exosomes or adeno-associated viruses, which increase the complexity of replacement therapies.

The critical contribution of the phenotypic changes in the neural, immune, and vascular cells in the pathophysiology of the SCI and the capabilities of miRNAs to modulate these changes (see previous sections) make miRNA therapeutics a highly promising approach to be explored. Although no microRNA-based therapy has entered clinical trials for SCI up to now, preclinical assays provide the necessary proof of concept. Experimental treatments with anti-miRs have demonstrated that inhibition of specific dysregulated microRNAs back to pre-injury levels can effectively reduce cell death. For example, Jee et al. (2012b) showed that local infusion of miR-20a inhibitor in the injured spinal cord reduced the expression of pro-apoptotic genes, and promoted neuron survival and functional recovery. Similarly, Hu et al. (2013b) also employed microRNA inhibition to explore the neuroprotective role of miR-21. Intrathecal infusion of miR-21 antagomir resulted in over-expression of pro-apoptotic genes, increased cell death and reduced recovery of the hindlimb motor function. On the other hand, two articles have proven that miRNA replacement can be also a viable therapeutic approach. Willemen et al. (2012) showed that intrathecal administration of miR-124 prevents persistent pain in rats, probably due to its modulation of microglial activation (Ponomarev et al., 2011). Im et al. (2012) reported similar results infusing miR-23b intrathecally to a murine model of neuropathic pain. According to these authors, restoring the normal levels of miR-23b reduced the expression of inflammatory proteins, particularly NOX4 in GABAergic neurons, protecting them from cell death, and ameliorating the neuropathic pain derived from SCI. These preclinical data strongly supports the feasibility of microRNA-based therapeutics in SCI treatment, although key aspects –including timing and side effects – remain to be elucidated. Detailed analysis characterizing the beneficial effects and determining the underlying mechanisms are strongly needed before microRNAs can reach the clinic in the treatment of the SCI.

Another clinically relevant but yet unexplored topic concerns how SCI alters the profiles of circulating microRNAs, and how information on these alterations can be used for diagnostic and prognostic purposes. Circulating or cell-free microRNAs are released to the body fluids either actively by secretion in exosomes or microvesicles or in association with RNA-binding proteins such as AGO2 and HDL; or passively, within apoptotic bodies liberated from dying cells (Chen et al., 2012; Zampetaki and Mayr, 2012). Encapsulation within lipid vesicles or association to binding proteins confers high stability to circulating microRNAs, despite the presence of large amounts of RNase in the body fluids (Chen et al., 2012; Li et al., 2012). Stability of circulating microRNAs together with the changes in microRNA expression in pathological states make circulating microRNAs promising biomarkers. In fact, since their discovery in all body fluids in 2008, nearly 500 articles have proposed several circulating microRNAs as biomarkers for different pathologies, including cancer, cardiovascular pathologies, and CNS injuries, among others (Laterza et al., 2009; Kosaka et al., 2010; Zhang et al., 2011; Pritchard et al., 2012). For example, analysis of the expression of circulating microRNAs after a traumatic brain injury has identified several potential biomarkers in humans (Redell et al., 2010) and rats (Balakathiresan et al., 2012). Also, it has been shown that the plasma concentration of neuron marker miR-124 becomes significantly increased after acute stroke (Laterza et al., 2009). However, quantification of circulating microRNAs can be challenging due to (i) their low concentrations, (ii) the effects of cell contaminants, and iii) the absence of endogenous controls for normalization. Low concentrations of circulating microRNAs suppose a technical challenge for microRNA extraction and quantification, and also increase the risk that microRNAs from contaminant cells, which are at much higher concentrations, would mask or confound the circulating microRNA profile (Kirschner et al., 2011).

Circulating microRNAs are also highly interesting due to their possible role as paracrine regulators. Circulating microRNAs can be transferred to neighboring or distant cells, altering the expression of target genes and regulating various functions, including proliferation, death or even tumor cell invasion (see Zhu and Fan, 2011, and references therein). Interestingly, microRNA transfer occurs when microRNAs are wrapped in exosomes, microvesicles, or apoptotic bodies, as well as when packaged with proteins (Zhu and Fan, 2011). Although most available evidence deals with circulating microRNA transfer in immune and vascular cells, a recent article has also demonstrated that miRNA transfer occurs between neural cells. According to Morel et al. (2013), neurons are able to secrete exosomes containing miR-124a, which are internalized by astrocytes causing an increase in the glutamate transporter GLT1.

## CONCLUDING REMARKS

Spinal cord injury is a complex pathology that induces strong cellular and molecular changes in the nervous, immune, and vascular systems. These changes alter the expression of the microRNAs -small non-coding RNAs that post-transcriptionally regulate the expression of thousands of genes- to different degrees up to a general downregulation of the microRNA expression. Bioinformatic analyses of the microRNA and mRNA expression profiles in the injured spinal cord have predicted that microRNA dysregulation strongly affects processes developing after the SCI. However, much more research analyzing the expression of specific cell populations and evaluating the effects of microRNA dysregulation is still needed if we want to validate the bioinformatic predictions, and to precisely characterize the changes in microRNA expression after SCI as well as their causes and their functional consequences. The pioneering studies developed up to now have been able to demonstrate the active role of individual microRNAs in the regulation of key processes of the SCI, such as cell death, inflammation, and astrogliosis. These results strongly suggest that microRNAs can be highly valuable therapeutic targets to modulate the deleterious events that follow SCI and to promote regenerative responses that will contribute to reduce the functional deficits associated to the SCI.

## AUTHOR CONTRIBUTIONS

All authors contributed in the conception and design of the present review, as well as in drafting and revising the manuscript. All authors have given full approval to the present version for its publication.

## Conflict of Interest Statement

The authors declare that the research was conducted in the absence of any commercial or financial relationships that could be construed as a potential conflict of interest.
